# A review of the presentation and outcome of left ventricular thrombus in coronavirus disease 2019 infection

**Published:** 2021-11-06

**Authors:** Anil Mathew Philip, Lina James George, Kevin John John, Anu Anna George, Jemimah Nayar, Kamal Kant Sahu, Vijairam Selvaraj, Amos Lal, Ajay Kumar Mishra

**Affiliations:** ^1^Department of Medicine, St. Thomas Mission Hospital, Kattanam, India; ^2^Department of Pulmonary Medicine, DR KM Cherian Institute of Medical Sciences, Kallissery, India; ^3^Department of Critical Care, Believers Church Medical College Hospital, Thiruvalla, Kerala, India; ^4^Department of Internal Medicine, Saint Vincent Hospital, Worcester, Massachusetts, 01608, USA; ^5^Department of Nuclear Medicine, Christian Medical College, Vellore, India; ^6^Division of Hematooncology, Huntsman Cancer Institute, University of Utah, United States; ^7^Internal Medicine, Warren Apert School of Brown University, Miriam Hospital, 164 Summit Ave, Providence, 02906, RI; ^8^Department of Medicine, Division of Pulmonary and Critical Care Medicine, Mayo Clinic, 200 1^st^ St SW, Rochester, MN 55905, United States; ^9^Department of Internal Medicine, Division of Cardiology, Saint Vincent Hospital, Worcester, Massachusetts, 01608, USA

**Keywords:** coronavirus disease 2019, severe acute respiratory syndrome, coronavirus 2, coronavirus, left ventricular thrombus, acute coronary syndrome, thrombosis, thromboembolism, echocardiogram

## Abstract

**Background:**

Cardiovascular complications of the coronavirus disease 2019 (COVID-19), which is caused by the severe acute respiratory syndrome coronavirus 2 (SARS- CoV-2), have been documented both in the acute phase and in convalescence. One such complication is the formation of the left ventricular (LV) thrombus. There is a lack of clarity regarding the incidence, risk factors, and management of this complication.

**Aim:**

The aim of the study is to identify the clinical presentation, risk factors and outcome of COVID-19 patients with left ventricular thrombus (LVT).

**Methods:**

A literature search was conducted to identify all case reports of COVID-19 with LVT in PubMed/Medline, Embase, Web of Science, and Google Scholar.

**Results:**

Among the 65 patients identified, 60 had LVT, either at admission, or during the acute phase of the illness. Six patients with mild symptoms during the acute phase of viral illness had only the COVID-19 antibody test positivity at the time LV thrombus was detected. Few of the patients (23.1%) had no comorbidities. The mean age of the patients was 52.8 years, and the youngest patient was 4 years old. This suggests that LVT formation can occur in young COVID-19 patients with no co-morbid conditions. Most of the patients (69.2%) had more than one site of thrombosis. A mortality rate of 23.1% was observed in our review, and ST-elevation myocardial infarction (STEMI) was diagnosed in 33.3% of those who died.

**Conclusions:**

A high degree of suspicion for LVT must be maintained in patients with known cardiac disease and those with new-onset arterial or venous thromboembolism, and such patients may benefit from a screening echocardiography at admission.

**Relevance for Patients:**

The patients with preexisting cardiovascular disease must take added precautions to prevent acquiring COVID-19 infection as there is a higher risk of developing LV thrombus. In patients who develop LVT in COVID-19, mortality rate is higher.

## 1. Introduction

The severe acute respiratory syndrome coronavirus 2 (SARS-CoV-2) or the novel coronavirus-2019 (nCoV-19), which was identified from Wuhan, China, is responsible for the coronavirus disease 2019 (COVID-19), which has caused nearly 4.3 million deaths worldwide. According to a meta-analysis from China, pre-existing cardiovascular disease is a predisposing factor for higher morbidity and mortality in COVID-19 [1]. Although acute respiratory failure and sepsis have been reported as the leading causes of death in COVID-19, direct and indirect cardiovascular complications such as myocardial injury, arrhythmia, acute coronary syndrome, and thromboembolism may also mortality. The mechanism of cardiac injury in COVID-19 may be ischemic or non-ischemic [2]. Thromboembolic complications are increasingly being reported as a complication of COVID-19 infection. The activation of endothelial cells by viral particles is thought to be the primary mechanism for thrombus development. The incidence of thrombosis in hospitalized non-critically ill patients is approximately 2.6% and is higher in critically ill patients (35.3%) [3]. During the pandemic the incidence of left ventricular thrombus (LVT) among patients presenting with myocardial infarction, it has reportedly increased [4]. This review aims to study the clinical presentations, risk factors, and outcomes of patients with COVID-19 who had presented with an LVT.

## 2. Methods

### 2.1. Eligibility criteria

This study included all patients with COVID-19 with LVT during any stage of illness. Case reports, mini-reports, and case series with individual patient details were pooled to assess clinical manifestations, imaging features, laboratory investigations, and outcomes. The diagnosis of COVID-19 was based on microbiological, radiology or serological tests.

### 2.2. Selection strategy

**Figure 1:**
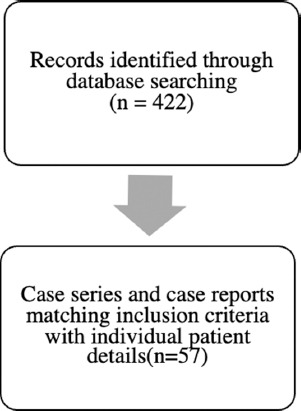
PRISMA flow chart of search

This review included articles on COVID-19 and LVT published in PubMed, Medline, Embase, Web of Science, and Google scholar till 16^th^ August 2021. The search terms used in the MeSH database, Web of science research assistant and Embase search were ‘“COVID-19” and “LVT”, “2019 nCoV” and “LVT”, and “SARS-CoV-2” and “LVT. In Google scholar, the terms “COVID-19 and LVT” were used and articles were sorted by relevance from 2019 onward. A total of 422 articles were identified. After eliminating duplicate articles, non-English articles and case reports with intracardiac thrombus at sites other than the left ventricle, a total of 62 articles (both case series and case reports) were identified. Five articles were eliminated because the diagnosis of COVID-19 could not be made with certainty. Authors were contacted to clarify aspects of the case reports that were vague. Full text was available for all 57 mansucripts that were finally included in the review. Data from all case reports and case series were pooled and analyzed. The findings of this analysis were compared to the other studies reported in the literature (Figure 1). Two independent clinicians were involved in the screening of the articles.

## 3. Results

Case reports and case series of a total of 65 COVID-19 patients with LVT were identified. Among them, 66.2% were male and the mean age was 52.8 years. Most of the cases were reported from the United States of America (USA).

### 3.1. Clinical features

The diagnosis of COVID-19 was made by reverse transcriptase-polymerase chain reaction (RT-PCR) of nasopharyngeal swab in 35 patients (53.8%), PCR of myocardial biopsy sample in one patient (1.5%), SARS CoV2 antibodies in six patients (9.2%), SARS CoV-2 rapid antigen test in one (1.5%) and computed tomography (CT) of the thorax in two patients (3.1%). The initial presenting symptom was dyspnea in 42 patients (64.6%), fever in 29 (44.6%), cough in 29 (44.6%), chest pain in 16 patients (24.6%), gastrointestinal symptoms such as vomiting and diarrhoea in six patients (9.2%), and neurological deficits in seven (10.7%). Twenty patients (30.8%) presented with hypoxia, one of whom was in shock, and COVID-19 pneumonia was present in 44 patients (67.7%). In 20 patients (30.8%), the mode of diagnosis of COVID-19 was not specified. The diagnosis of LVT was made at the time of admission in 37 patients (56.9%). Among the rest, LVT was detected between days one and 30 of hospital admission.

### 3.2. Risk factors

The most common comorbidity seen was pre-existing heart disease which was present in 14 (21.5%) patients, wherein chronic heart failure with reduced ejection fraction and pre-existing coronary artery disease was observed in eight (12.3%) and six (9.2%) patients, respectively. Diabetes mellitus, hypertension, dyslipidemia, obesity, and obstructive airway disease were seen in eleven (16.9%), nine (13.8%), six (9.2%), four (6.1%), and eight (12.3%) patients, respectively. Fifteen patients (23.1%) had no comorbidities, and in eight patients (12.3%) comorbidities were not reported.

### 3.3. Lab reports and imaging

C-reactive protein (CRP) was reported in 29 patients (44.6%) and was elevated (>10 mg/L) in 27 of them (41.5%). Troponin was reported in 34 patients (52.3%) at admission and was elevated (>0.14 μg/L) in 22 (33.8%). D-dimer was elevated (>500 ng/ml fibrinogen equivalent units) in 29 (44.6%) of the 35 patients for whom it was reported, and 23 patients (35.4%) had elevated brain natriuretic peptide.

Electrocardiogram (ECG) was reported in 39 patients (60%). Fourteen of them (21.5%) had ST-segment changes at admission (Table 1). Nineteen of them (29.2%) underwent coronary angiography (CAG), seven of whom (10.8%) had thrombi in either the right coronary artery or left anterior descending artery. Lung involvement by COVID-19 was present in 45 patients (69.2%), which was demonstrated by either chest roentgenogram (X-ray), or CT thorax, or both. CT pulmonary angiogram showed pulmonary embolism in 11 patients (16.9%), four of whom had right ventricular thrombus as well. One patient had the presence of inferior vena caval, renal, and iliac vessel thrombosis [5]. The presence of LVT was identified by 2D echocardiogram in 40 patients (61.5%), CT thorax in twelve patients (18.5%), cardiac magnetic resonance imaging (MRI) in nine patients (13.8%), and ventriculography in one patient (1.5%). Apical thrombus was present in 36 patients (55.4%), while the site of thrombus was not specified in 29 patients. Eight patients (12.3%) had ventricular aneurysms, and five had biventricular thrombi (7.7%). LV dysfunction and reduced ejection fraction were noted in 44 patients (67.7%).

**Table 1:** Summary of laboratory findings in COVID-19 patients with the left ventricular thrombus

	Reference	BNP (ng/ml)	CRP (mg/L)	Troponin T (mcg/L)	D-dimer (ng/ml)
1	Mahdavi [57]	8827	No data	No data	No data
2	Bigdelian [58]	No data	11	No data	2000
3	Bigdelian [58]	No data	29	No data	490
4	Materna [59]	Elevated	Elevated	Elevated	Elevated
5	Schroder [60]	17950	301	2.18	3800
6	Munoz [61]	34	No data	0.12	390
7	Agarwal [62]	5166	Normal	1.326	2630
8	Iguina [8]	1000	No data	0.34	856
9	Hodson [62]	No data	No data	No data	No data
10	Capaccione [64]	No data	No data	No data	500
11	Ranard [64]	315	5.96	0.386	500
12	Kihira [66]	No data	No data	No data	700
13	Ziaie [11]	23000	103	No data	1350000
14	Amin [67]	No data	No data	No data	No data
15	El Aidouni [68]	No data	114	0.1	No data
16	Paolo Rubartelli [69]	1702	No data	0.116	5004
17	Jadhav [70]	No data	No data	No data	No data
18	Jariwala [71]	No data	No data	Elevated	2322
19	Hammam [72]	2215	147	0.734	3.4
20	Ceci Bonello [73]	No data	115	0.504	5483
21	Garg [74]	No data	118	0.91	8100
22	Hudowenz [75]	12,232	130	3.264	No data
23	Jadhav [70]	5080	No data	1.81	No data
24	Mitevska [9]	6700	45	No data	No data
25	Venkataraman Pranav [76]	No data	50	0.181	No data
26	Alizadehasl [787]	No data	No data	No data	No data
27	Sharma [78]	No data	374	2.54	No data
28	Ramalho [79]	30.39	641	0.628	No data
29	Gravinay [80]	900	270	2900	
30	Ford [81]	588	No data	0.066	No data
31	Jariwala [71]	No data	No data	No data	7809
32	Servato [82]	l7460	123.5	low	low
33	Jeon [5]	No data	No data	No data	No data
34	Imaeda [83]	683.4	22.9	0.028	3000
35	Malaweera [84]	No data	No data	No data	7480
36	Jariwala [85]	No data	No data	No data	6548
37	Zheng [86]	11,463	No data	0.05	na
38	Ferguson [10]	No data	405	No data	508
39	John [87]	No data	No data	0.01	629
40	Ozer [88]	34824	197	0.05	20000
41	Farouji [89]	517	144	negative	64,000
42	Tadayoni [90]	No data	No data	No data	No data
43	Byer [91]	No data	No data	No data	No data
44	Soltani [92]	No data	No data	937	No data
45	M Ignaszewski [93]	No data	No data	No data	No data
46	Jadhav [70]	No data	No data	No data	No data
47	Jadhav [70]	No data	No data	No data	No data
48	Iqbal Phool [94]	No data	170.4	0.189	3370
49	Jariwala [721]	No data	No data	Elevated	4566
50	Singh [95]	No data	No data	No data	4558
51	Castro [96]	No data	164	No data	No data
52	Mandal [97]	No data	54	No data	No data
53	Calvi [91]	No data	No data	No data	No data
54	Nanthatanti [98]	No data	No data	No data	1548
55	Azhar [99]	>20000	No data	0.04	No data
58	Meriem Boui [100]	No data	115	2.45	11200
57	Gozgec [101]	No data	No data	No data	No data
58	Bernardi [7]	8999	14.2	0.775	2931
59	Calvi [91]	No data	No data	No data	2931
60	Furtney Joshua [102]	No data	No data	No data	No data
61	Alfaki [103]	1830	No data	6.89	>20.00
62	Patell [6]	No data	No data	No data	No data
63	Sonaglioni [104]	>20,000	111	0.08	17,108
64	Fenton [105]	No data	412	0.354	No data
65	Jariwala [71]	No data	No data	No data	13453

BNP: Brain natriuretic peptide; CRP: C- reactive protein

### 3.4. Outcome

More than one site of thrombus formation was noted in 45 (69.2%) patients. Stroke was a complication in 14 patients (21.5%), acute coronary syndrome in 15 (23.1%), pulmonary embolism in 11 (16.9%), and peripheral arterial embolism in eight (12.3%) (Table 2). One patient had central retinal artery occlusion [6], and three had peripheral venous embolism (4.6%). Four patients (6.2%) had renal infarcts, among whom two had splenic infarction as well. Isolated splenic infarction was seen in two patients (3.1%). The cause of thrombosis could be attributed to an acute coronary event in 15 patients (23.1%). MRI confirmed viral myocarditis in eight patients (12.3%) and one patient developed takotsubo cardiomyopathy [7]. Thrombophilia workup showed anti-phosphatidyl serine antibodies in one patient [8], and the presence of heterozygous mutations for Factor V Leiden, prothrombin and PAI-1 antibodies in another [9] Heparin-induced thrombocytopenia (HIT) was suspected in one patient; however, antibodies were negative [10]. One patient who developed LVT was later diagnosed with hyper-eosinophilic syndrome [11]. Three patients (4.6%) with LVT had no previous or ongoing cardiac or coagulation abnormalities. All patients were treated with low molecular weight heparin, which was later modified to coumarins or novel oral anticoagulants. The overall mortality was 23.1%. Five of the 15 patients who died had ST-elevation on ECG, either during diagnosis or during the course of hospital admission. All patients who died were hypoxic at admission or immediately after. Among the 50 patients who survived, follow-up data was available for 30, all of whom had a decreased or complete dissolution of the thrombus. Five patients underwent thrombus extraction by either CAG guided thrombolysis with peripheral extraction, or surgical LV thrombectomy, and had no recurrence during follow-up.

**Table 2:** Outcomes of patients presenting with left ventricular thrombus

Outcome	*n* (%)
Myocardial infarction	15 (23.1)
New onset heart failure	16 (24.6)
Myocarditis	8 (12.3)
Takotsubo cardiomyopathy	1 (1.5)
Biventricular thrombus	5 (7.7)
Cerebrovascular accident	14 (21.5)
Peripheral arterial embolism	8 (12.3)
Deep vein thrombosis	5 (7.7)
Pulmonary embolism	11 (16.9%)
Organ involvement	
Renal	4 (6.2)
Splenic	4 (6.2)
Hepatic	1 (1.5)
Eye	1 (1.5)

## 4. Discussion

Among the underlying etiologies for LVT formation, the most common was dilated cardiomyopathy followed by myocardial infarction [12]. Both right and LVT formation have been reported in patients with COVID-19, with the latter being more common. A study conducted among 3334 hospitalized patients with COVID-19 in New York showed that the incidence of thrombotic complications was 16%, 11% of which were arterial thrombosis, and 6.2% were venous [13]. This is higher than what was observed during the influenza pandemic of 2009, when the overall incidence of thrombotic complications was 5.9% [14]. The risk of myocarditis was higher in COVID-19 when compared to influenza (Table 3) [15]. In a Danish nationwide study which followed-up COVID-19 cases, the incidence of myocardial infarction was 5 times higher in the 14 days following the diagnosis of COVID-19, compared to 180 days prior [16].

**Table 3:** Summary of case reports of patients with COVID-19 with the left ventricular thrombus

	Reference	Age	Sex	Comorbidities	Diagnosis	Mode of diagnosis	Resolution of thrombus
1	Mahdavi [57]	4	F	Nil	LV thrombus, myocarditis	MRI	Expired
2	Bigdelian [58]	8	F	Orthopedic surgery few weeks back	Biventricular thrombus, pulmonary embolism	Echo	Surgical thrombectomy
3	Bigdelian [58]	11	F	Nil	Biventricular thrombus	Echo	Surgical thrombectomy
4	Materna [59]	17	M	Nil	LV thrombus with CVA	Echo	Extracted, no recurrence. LV function normalized in 42 hrs
5	Schroder [60]	17	M	Nil	LV thrombus, MIS-C	Echo	9 days
6	Munoz [61]	18	M	Nil	Myocarditis, LV thrombus	Echo followed by MRI	Non-compliant with treatment, persistent at several months
7	Agarwal [62]	26	M	Nil	Biventricular thrombus, non-obstructive MI, new onset heart failure	Echo followed by MRI	12 days
8	Iguina [8]	27	F	DM, PCOS on OCP	LV thrombus with CVA and APS antibodies	Echo	Not known
9	Hodson [63]	29	M	asthma	Myopericarditis and LV thrombus	Echo followed by MRI	No data
10	Capaccione [64]	35	M	intermittent asthma, mild obesity	CVA, NSTEMI, LV thrombus	Echo	6 days
11	Ranard [65]	35	M	asthma, obesity	CVA, NSTEMI, LV thrombus	Echo followed by MRI	Reduced size at 6 days
12	Kihira [66]	37	M	Nil	LV thrombus with CVA	Echo	Not known
13	Ziaie [11]	39	F	Asthma	LV thrombus with HES	Echo	8 days
14	Amin [67]	39	F	Asthma	LV thrombus, myocarditis	Echo	3 days
15	El Aidouni [68]	40	F	Psychosis	LV thrombus, DVT peripheral arterial embolism	Echo	Not known
16	Paolo Rubartelli [69]	43	M	HfrEF, EF=48	Myocarditis, LV thrombus, pulmonary artery DVT, IVC thrombus, renal infarct	CT Angiogram followed by echo	More than 4 months
17	Jadhav [70]	43	M	Not known	IWMI, LV thrombus	Echo	Persistent thrombus at 20 days
18	Jariwala [71]	45	M	DM, smoker	LV thrombus with STEMI, homocysteinemia	Echo followed by MRI	30 days
19	Hammam [72]	47	F	Nil	LV thrombus peripheral arterial thrombus, DVT	Echo	30 days, LVEF improved to 30%
20	Ceci Bonello [73]	47	M	DM, DLP	LV thrombus with CVA, splenic, renal infarct, peripheral arterial infarcts	Echo	Surgical thrombectomy
21	Garg [74]	48	F	HTN	STEMI, LV thrombus, CVA	Echo	Expired
22	Hudowenz [75]	48	M	Asthma	Myocarditis, LV thrombus	MRI	90 days
23	Jadhav [70]	48	F	DM, HTN	AWMI, LV thrombus	Echo	15 days
24	Mitevska [9]	48	M	Nil	Biventricular thrombus, pulmonary embolism, DVT	Echo	Not known
25	Venkataraman Pranav [76]	49	M	No data	STEMI, LV thrombus	Echo	Not known
26	Alizadehasl [77]	49	F	Nil	LV thrombus	Echo	No details
27	Sharma [78]	50	M	DM, DLP in admission	STEMI, LV thrombus, peripheral arterial thrombus	Echo	Expired
28	Ramalho [79]	50	M	DM, DLP	new onset DCM, LV thrombus	Echo	2 months
29	Gravinay [80]	51	M	No data	Myocarditis, LV thrombus	MRI	No data
30	Ford [81]	53	M	DLP	CVA with LV thrombus and myocarditis, possible chagas	Echo	Not known
31	Jariwala [71]	54	M	DM, smoker	LV thrombus with STEMI	Echo	LV thrombectomy done
32	Servato [82]	55	M	Obesity, OSA on CPAP	LV thrombus, myocarditis	Echo	7 days
33	Jeon [5]	55	M	Not known	LV, pulmonary embolism, liver, kidney, spleen	Echo	1 month
34	Imaeda [83]	56	M	DCM. EF-30	LV thrombus	CTPA followed by echo confirmation	8 days
35	Malaweera [84]	56	M	CAD, LV thrombus received 1yr anticoagulation	LV thrombus, spontaneous pneumothorax, pulmonary embolism	CTPA	Not known
36	Jariwala [85]	56	M	Chronic pancreatitis	LV thrombus	CTPA	Expired
37	Zheng [86]	57	M	DM, HTN, non-ischemic DCM, rEF	CVA, LV thrombus	Echo	Not known
38	Ferguson [10]	58	M	HTN, obesity, previous smoker	Biventricular thrombus, pulmonary embolism, peripheral arterial thrombosis	CTPA	Not known
39	John [87]	58	M	Nil	LV thrombus with STEMI	Echo	6 weeks, normal EF at 5 months
40	Ozer [88]	58	M	DM, HTN	Biventricular thrombi, DVT, myocarditis	CTPA	Pt expired
41	Farouji [89]	60	M	HFrEF, epilepsy, and schizophrenia, active smoker,	LV thrombus, pulmonary embolism	CTPA followed by echo confirmation	Reduced size at 6 weeks
42	Tadayoni [90]	61	M	HOCM, LV aneurysm, GBS post COVID	LV thrombus, GBS	Echo	Not known
43	Byer [91]	62	F	Ischemic DCM	LV thrombus,	Echo	Not known
44	Soltani [92]	63	F	Smoker, emphysema	Biventricular thrombi, pulmonary embolism, STEMI	Ventriculography followed by cardiac CT	Expired
45	Ignaszewski [93]	63	M	Nil	STEMI, LV thrombus, HF	Echo followed by MRI	Not known
46	Jadhav [70]	63	M	No data	AWMI, LV thrombus, CVA	Echo	Expired
47	Jadhav [70]	64	F	No data	LV thrombus with CVA	Echo	Expired
48	Iqbal Phool [94]	65	M	Nil	CVA, LV thrombus	Echo	1 month
49	Jariwala [71]	67	M	DM, reformed smoker	LV thrombus with STEMI	Echo	2 weeks
50	Singh [95]	69	F	Pulmonary embolism on apixaban	LV thrombus with CVA	Echo	Expired
51	Castro [96]	70	F	HTN	LV thrombus	Echo	Expired
52	Mandal [97]	70	F	CAD s/p CABG and LV aneurysm resection, COPD	LV thrombus with splenic infarct, peripheral arterial infarcts	CTPA	Not known
53	Calvi [91]	70	M	CAD, HFrEF 33%, Lt pneumonectomy for adenocarcinoma lung	LV thrombus, VA, splenic infarct	Echo followed by CT	12 days
54	Nanthatanti [98]	71	M	HTN, DLP, CAD	LV thrombus,	CTPA followed by echo confirmation	Not known
55	Azhar [99]	71	F	Not known	LV thrombus, DVT pulmonary embolism	CT angiogram followed by echo	Not known
56	Boui [100]	73	M	Gout	LV thrombus, pulmonary embolism, renal thrombus	CTPA followed by echo confirmation	55 days
57	Gozgec [101]	74	F	Nil	LV thrombus,	CT	Expired
58	Bernardi [7]	74	M	DM, HTN, DLP	LV thrombus with Takotsubo syndrome	Echo followed by MRI	14 days
59	Calvi [91]	74	M	Nil	LV thrombus,	Echo followed by MRI	13 days
60	Furtney Joshua [102]	78	F	Not known	LV thrombus	Echo	Not known
61	Alfaki [103]	79	M	Non-ischemic cardiomyopathy, EF-45-50	LV thrombus, pulmonary embolism	Echo	Expired
62	Patell [6]	80	F	Not known	LV thrombus with CRAO	Echo	Not known
63	Sonaglioni [104]	80	F	CAD, HFrEF, CKD	Biventricular thrombus	Echo	Expired
64	Fenton [105]	82	M	Nil, smoking history	STEMI, LV thrombus	Echo	Expired
65	Jariwala [71]	85	M	HTN, CAD,	LV thrombus, CVA, carotid artery thrombus	Echo	Expired

CABG: Coronary artery bypass grafting, CAD: Coronary artery disease, CAG: Coronary angiography, CMR: Cardiac magnetic resonance imaging, CRAO: Central retinal artery occlusion,

CT: Computed tomography, CTPA: Computed tomography with pulmonary angiogram, CVA: Cerebrovascular accident, DCM: Dilated cardiomyopathy, DLP: Dyslipidemia, DM: Diabetes mellitus, DVT: Deep vein thrombosis, HFrEF: Heart failure with reduced Ejection fraction, HTN: Hypertension, LV: Left ventricle, MRI: Magnetic resonance imaging, NSTEMI: Non-ST segment elevation myocardial infarction, OSA: Obstructive sleep apnea, PTCA: Percutaneous transluminal coronary angioplasty, STEMI: ST segment elevation myocardial infarction

Since the rates of LVT in acute myocardial infarction and acute idiopathic myocarditis were 45% and 61.9%, respectively, a similar or higher incidence can be expected in COVID-19 [17]. This is supported by the observation that patients with concomitant COVID-19 and STEMI had worse left ventricular function, myocardial blush grade, higher incidence of multivessel disease, and stent thrombosis when compared to non-COVID-19 patients [18,19]. STEMI was the initial presentation in 69.2% of the patients with COVID-19, in an Egyptian study of 26 patients [19]. In our review, symptoms of typical anginal pain, as the presenting symptom were noted in nine patients (31%).

Post-infarct complications can lead to severe morbidity and mortality in these patients; however, the mortality from acute myocardial infarction has decreased after the incidence of percutaneous coronary intervention (PCI). The incidence of LVT post-acute myocardial infarction was 17% in the pre-PCI era, with an incidence as high as 34% in patients with anterior MI [20]. After the introduction of primary PCI, the incidence of LVT has fallen drastically, with incidence rates as low as 1.6% [21]. However, meta-analyses have found the rates to be between 7.5 and 9.1% in anterior MI [22].

Higher rates of thrombus formation were found in patients with anterior MI, low ejection fraction, severe apical wall motion abnormality, and worse TIMI flow rates.

### 4.1. Pathogenesis

The incidence of thrombotic complications may be higher in patients diagnosed with COVID-19 due to one of the following reasons.

1. The direct effects of the SARS-CoV2 virus, such as inhibition of interferon production and cytopathic effects on the CD4 cells leading to CD4+ lymphopenia, may stimulate downstream activation of proinflammatory macrophages and polymorphs, resulting in the release of prothrombotic cytokines and activation of platelets [23].
2. Infections such as COVID-19 can stimulate inflammatory activity inside an atheromatous plaque by activating macrophages and T-cells leading to a disruption of the plaque surface, exposure of its underlying thrombogenic elements and the formation of a thrombus [24].
3. Direct effects on the myocardium and COVID-19 induced myocarditis have been reported in 1% of all hospital admission [25]. In autopsy specimens, cardiac injury was noted in as many as 35%, with 13% showing lymphocytic myocarditis [26].
4. COVID-induced hypoxia may contribute to increased circulatory demand in the form of a compensatory increase in heart rate to maintain tissue oxygenation. Sustained hypoxia can lead to an increased production of transcription factors such as Nuclear Factor-kb and Hypoxia-inducible Factor-1, leading to further the inflammatory cascade and thrombosis [27].
5. COVID-19 has been shown to stimulate the production of neutrophil extracellular traps, which contribute to an increased risk of microvascular and venous thrombus formation [28]. In neutrophils incubated with the SARS-CoV2, increased levels of reactive oxygen species and serum levels of cell-free DNA, myeloperoxidase-DNA, and citrullinated histone H3 have been seen [29]. The virus exhibits tropism for angiotensin-converting enzyme -2 (ACE-2), which is found in type II epithelial cells of the lung, heart, kidneys, intestines, and blood vessels. In the heart, the receptor has been found on the pericytes, myocytes, and endothelial cells [30]. Higher concentrations of ACE- 2 may be found in the pericytes of patients with heart failure [31]. This may predispose these patients to a higher incidence of cardiac involvement. Direct cytopathic effects on the cardiac endothelial cells may be responsible for endothelial cell injury, apoptosis, and resultant thrombosis [32]. In response to viral replication, the host defence mechanism attempts to downregulate the levels of ACE-2 in the heart. This can, in turn, lead to an increase in the prothrombotic and proinflammatory effects of angiotensin II, leading to the formation of thrombosis and an increase in troponins, which in turn is associated with a poorer prognosis [33].
6. Prolonged hospitalization, ICU admission, and intubation are risk factors contributing to immobilization and venous stasis.
7. Antiphospholipid antibody syndrome is a prothrombotic autoimmune disease due to the presence of antiphospholipid antibodies, such as lupus anticoagulant (LA) anticardiolipin antibodies LA, or anti-b2glycoprotein-1. COVID-19 has been associated with detecting antiphospholipid antibodies in at least 52% of the patients [34]. The pathogenesis behind this finding can be attributed to molecular mimicry between the spike protein of the SARS CoV2 and native phospholipids, leading to the generation of antiphospholipid antibodies. Another possible mechanism is the conformational change in b2 glycoprotein induced by the oxidative stress in COVID-induced cytokine release, leading to a neoepitope formation and increased immunogenicity [35].
8. The increased incidence of Takotsubo cardiomyopathy in the post-COVID-19 period has been observed based on the results of a Cleveland clinic study, with rates as high as 7.7% in all patients with acute coronary syndrome compared to less than 2% before the pandemic [36,37]. This may contribute to the development of mural thrombus, as 3.3% of all patients with Takotsubo syndrome have been found to develop LVT [38].
9. COVID-19 associated HIT has been proven by the demonstration of elevated HIT antibodies against heparin-PF4 complexes. It leads to increased activation of the complement system, accumulation of C3a complement and increased arterial and venous thrombosis, especially in patients with severe COVID 19 [39].

### 4.2. Investigations

Echocardiography was the most commonly used imaging modality for detecting LVT. Early identification and treatment of patients with LVT are essential to improve outcomes. Transthoracic echocardiogram (TTE) is usually the initial modality used for evaluating LVT [40]. However, sensitivity is reportedly as low as 21%. Routine echocardiograms may oversee a small mural thrombus, especially when the clinical indication does not warrant a high degree of suspicion [41,42]. This can be improved with intravenous contrast, which raises sensitivity to approximately 64% [43]. Delayed enhanced cardiac MRI (CMR) is the gold standard for evaluating LVT, with a sensitivity of up to 88% and specificity of 99–100% verified by surgical findings, and with the highest detection rates when done 9–12 days after myocardial infarction [44]. Delayed enhancement CMR relies on tissue characterization to detect LVT rather than anatomic appearance, allowing thrombus to be differentiated from myocardial structures regardless of location or morphology [40]. Some studies have reported coronary CT angiography (CCTA) to be comparable to CMR in detecting LVT with advantages including shorter scanning time and widespread availability. Disadvantages of CCTA include increased radiation to patients and the need for intravenous iodinated contrast [45]. In one case study, persistent Staphylococcus bacteremia was detected in a 61-year-old woman with fever and acute meningitis. A transesophageal echocardiogram did not reveal any pathological findings. However, 18F-FDG PET/CT and CMR helped diagnose a left ventricular infected thrombus [46]. Among the modalities of diagnosis, contrast-MRI with late gadolinium enhancement yields accurate results with a sensitivity of 88% and specificity of 99%. This is followed by the cine CMR, contrast TTE, and non-contrast TTE, among the non-invasive methods. Although contrast ventriculography has a high specificity (85–90%), the sensitivity ranges to around 30%, especially immediately after a MI [47]. Fiberoptic cardioscopy is an endoscopic system developed in Japan, and is used to assess the morphology and functionality of the interior of the cardiac chambers and enable minimally invasive procedures [48]. Cardioscopy can detect LVT in 30.2% of the cases, compared to 2.7% with left ventriculography, 7.0% with contrast echocardiogram, and 1.9% with non-contrast echocardiography [49]. No direct study has compared the relative efficacy of cardioscopy over CMR. However, Uchida proposes that cardioscopy may be more valuable in this cause, as it is more sensitive (35.1% vs. 16.3%, *P*<0.01), and can also detect the characteristics of the thrombus, such as morphology and color. In the present pandemic, due to the need for sophisticated equipment and the ability to visualize only 4-5 sections of the LV, non-invasive diagnostic tests may be preferred for ease of access and availability.

### 4.3. Management

All patients with cardiovascular comorbidities, who receive medical care at home, should be closely assessed for disease worsening and need for hospitalization [50]. Patients who require hospitalization and those who require critical care should receive routine thromboprophylaxis. TTE is indicated in hospitalized patients with cardiac comorbidities or high clinical suspicion, especially those with raised troponins and D-dimer values. These correlate with a higher risk of detecting critical findings on the echocardiogram and may necessitate a change in treatment strategy [51,52].

In hospitalized patients, unfractionated heparin (UFH) or LMWH may be preferred over direct oral anticoagulants due to the lower risk of drug-interaction with antivirals or steroids. Using heparin in the form of UFH or LMWH improved the 28-day mortality in hospitalized patients with COVID-19, who had D-dimer above 3000 ng/ml [53,54]. Extended post-discharge thromboprophylaxis is recommended only in patients with a high risk of post-discharge thrombosis. In the pre-COVID-19 period, studies on LVT demonstrated that the median time to thrombus regression was 103 days, irrespective of the anticoagulant given [55]. There was no difference in the rate of embolic or bleeding events among the different anticoagulants [56].

## 5. Limitations

Most studies on COVID-19 and cardiac complications do not have data on the incidence of LVT; hence, the actual incidence in the real-world setting cannot be estimated. Although preliminary data suggests that there is an association between LVT and COVID-19, one cannot assume that the latter is the cause of the former, in the absence of unequivocal evidence from a large, prospective study. Studies with directed cardiac imaging and a high degree of suspicion are required in COVID-19 patients with documented peripheral arterial or venous embolism to differentiate between small vessel *de novo* thrombosis and embolism secondary to intra-cardiac thrombosis. Due to a lack of uniformity in clinical, imaging, and laboratory data between the patients included in our review, definite conclusions could not be drawn regarding any parameter that could be considered helpful in screening or assessing patients presenting with LVT. Details of outcomes including ARF, ARDS, CNS injury, mechanical ventilation, duration of stay, hemodialysis, and cost of care have not been reported.

## 6. Conclusion

Although LVT has high morbidity and mortality in patients with COVID-19, routine screening of patients is not required. With only a few cases reported, the clinical presentation and laboratory parameters necessitating screening for LVT are uncertain. LVT has been commonly reported in the COVID-19 patients with severe infection and underlying myocardial dysfunction. Cardiac screening studies may be necessary for COVID-19 patients with severe infection, elevated coagulation parameters, and comorbidities to rule out mural thrombus, and the possibility of arterial or pulmonary embolism. Bedside screening echocardiogram can be done in patients with preexistent cardiac illness, and moderate to severe COVID-19 at the time of admission.

Further imaging is warranted only if clinical or laboratory parameters suggest increasing severity of disease. Since COVID-19 pneumonia and resultant hypoxia have been associated with an increased risk of thrombosis, thromboprophylaxis must be initiated in all patients with moderate to severe disease. A high index of suspicion should also be maintained in patients presenting with arterial and venous embolism.
